# Supplementary material for: Examining the surface evolution of LaTiOxNy an oxynitride solar water splitting photocatalyst
Source: Nat Commun. 2020 Apr 7;11:1728. doi: 10.1038/s41467-020-15519-y (PMC7138824; doi:10.1038/s41467-020-15519-y)
Supplement: Supplementary file 1 — Supplementay Information [file 41467_2020_15519_MOESM1_ESM.pdf]

Supplementary Information

Examining the surface evolution of $LaTiO_xN_y$ an oxynitride solar water splitting photocatalyst

Lawley et al.

Supplementary Figures

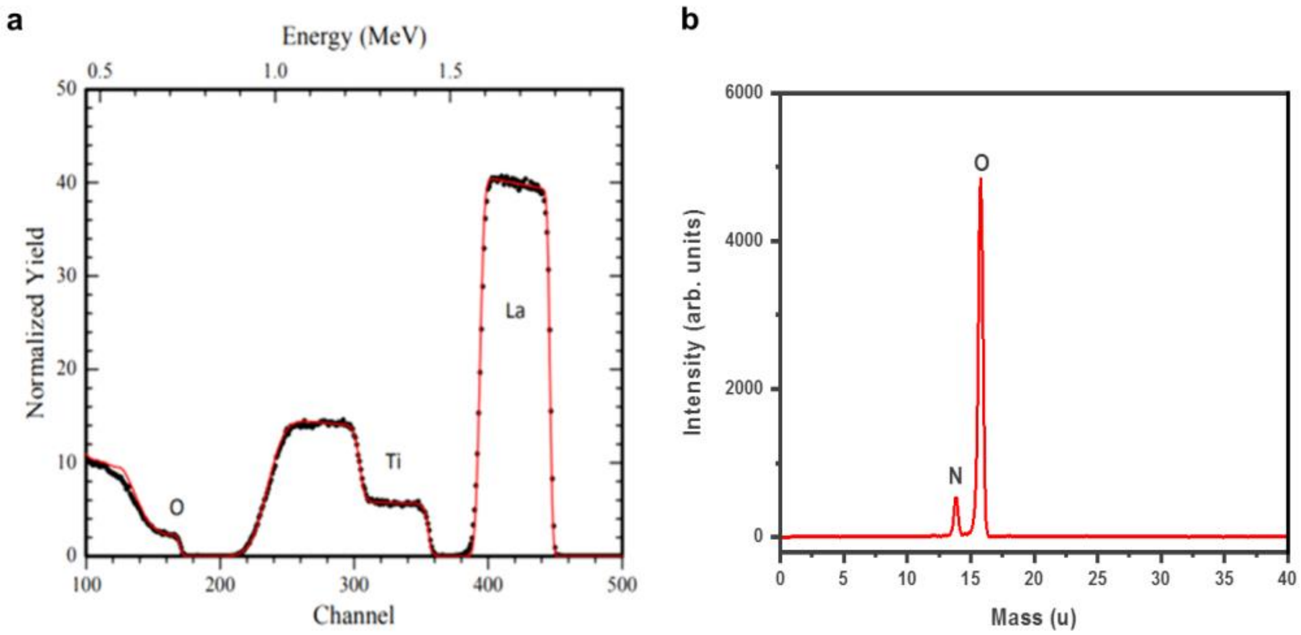

Supplementary Figure 1. Ion beam analysis of LTON. a, Rutherford backscattering (RBS) spectrum. **b,** elastic recoil detection analysis (ERDA) spectrum. The cation ratios were determined by RBS whereas, the O:N ratios were determined by ERDA

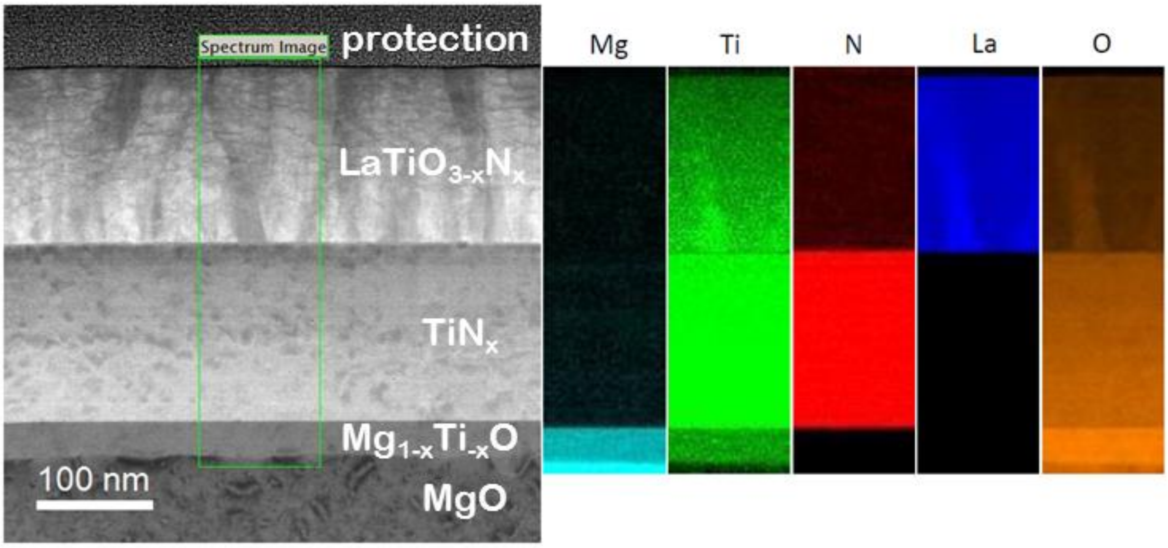

Supplementary Figure 2. Transmission Electron Microscopy (TEM) cross sectional image for a LTON film. The TEM cross image signifies there is an additional layer/interface at the substrate-TiN interface. Electron energy spectroscopy (EELS) was able to determine the presence of the elements and could determine that this additional layer comprised on Mg, Ti and O. During growth of the sample small amounts of Ti diffused into the MgO substrate and O diffused from the MgO substrate into the TiN layer.

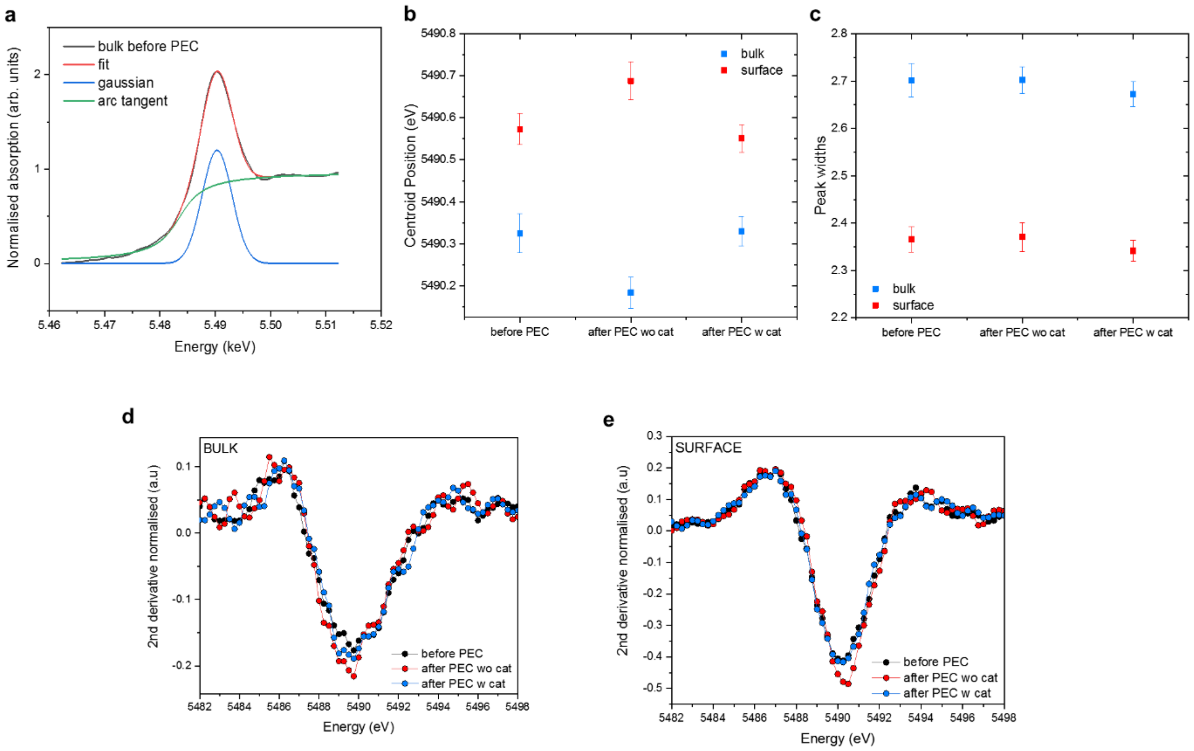

Supplementary Figure 3. La L_3 XANES peak fitting analysis. a, example of peak fitting for one of the La L_3 spectra. **b,** centroid position of peak for each spectra from figure 5b-c. **c,** peak FWHM. **d,** second derivative for bulk XANES measurements. **e,** second derivative for surface XANES measurements.

Supplementary Tables

Supplementary Table 1. Compositions of LTON thin films determined by RBS and ERDA.

Film	Composition	La:Ti	O:N
LTON sample batch 1	$La_{1.03}Ti_{0.97}O_{3.0}N_{0.29}$	1.06(0.02)	10.3(0.7)
LTON sample batch 2	$La_{1.02}Ti_{0.98}O_{3.0}N_{0.28}$	1.04(0.02)	10.7(0.7)

Supplementary Table 2. Neutron reflectometry layer fitting model

Before PEC

Parameter	Thickness(nm)	composition
LTON Surface	1.59	La1.03 Ti0.97 O2.7 N0.3
LTON bulk 1	28.40	La1.03 Ti0.97 O2.6 N0.4
LTON bulk 2	15.07	La1.03 Ti0.97 O2.5 N0.5
LTON bulk 3	58.17	La1.03 Ti0.97 O2.4 N0.6
TiON 2	12.09	Ti1 N 0.9 O0.1
TiN	87.69	Ti1 N1
TiON	19.00	Ti1 N0.95 O0.05

After PEC

Parameter	Thickness(nm)	composition
LTON Surface	4.49	La1.03 Ti0.97 O3
LTON bulk 1	5.34	La1.03 Ti 0.97 O2.9 N0.1
LTON bulk 2	37.50	La 1.03 Ti0.97 O2.8 N0.2
LTON bulk 3	49.26	La1.03 Ti0.97 O2.4 N0.6
LTON bulk 4	7.70	La1.03 Ti0.97 O2.42 N0.58
TiON 2	12.59	Ti1 N0.9 O0.1
TiN	87.89	Ti1 N1
TiON	18.71	Ti1 N0.95 O0.05

Supplementary Table 3. Pre edge peak positions and assignments for TiO2 and LTON. The ΔE of the centroid positions have been aligned by setting peak A1 to zero on the energy scale. Peaks C2 and C3 were not included in the differential peak fit model for LTON since there was no change in the pre edge intensity for those contributions. Peak B2's presence was only suggested for the LTON film.

Peaks	ΔE TiO_2	ΔE LTON	Peak assignment	orbitals
A1	0.0	0.0	3d-4p	B_{2g} , a_g
A2	1.6	1.7	3d-4p	b_{3g}
A3	3.0	3.0	3d-4p	a_g , b_{1g}
B1	5.4	4.5	4p-4s	a_g
B2	-	7.0	-	-
C1	9.9	9.2	$4p_z$	b_{1u}
C2	11.3	-	$4p_y$	b_{2u}
C3	14.3	-	$4p_x$	b_{3u}

Supplementary Discussion

Lanthanum L_3 XANES:

The centroid positions of the peak fits show that after PEC in the bulk La centroid position shifts to lower energy by ca. 0.1 eV. With the cocatalyst, the centroid position remains unchanged. At the surface, the opposite trend is observed where after PEC without the cocatalyst the centroid position shifts to higher energy ca. 0.15 eV. Again, in the presence of the cocatalyst this shift is prevented (within error). Interestingly the centroid positions follow opposite trends yet the FWHMs follow the same trend.

It can be seen that the peak widths in the bulk and surface measurements for LaTiO2N have FWHM ranges approximately between 2.65-2.75 and 2.3-2.4 respectively. However, comparing bulk vs surface is complicated since the bulk measurements suffer from dampening due to self-absorption in the thin film. The bulk measurements can be compared to one another performed at the same probe depth as the self absorption effect would be equal for each measurement.

It is also of note that the peak FWHMs do not change by very significant amounts and are also within error. Compared to[1], which looks at FWHM shifts of ca. 0.5 eV for differently coordinated species. Therefore although interesting, this approach may not be as useful/accurate enough as suggested to compare the coordination of La to further our understanding.
A second study[2] looked at the white line intensity with respect to symmetry/disorder effects by changing coordination. They note differences in the second derivatives between species due to changing coordination and degree of disorder of the La octahedral by looking at symmetry with respect to the t2g and eg splitting.

From the LaTiOxNy measurements it can be seen there are no significant changes again in the second derivatives plots after PEC without a catalyst. There is no evidence of strong disorder or symmetry changes of La at the surface or bulk before and after PEC just centroid position shifts in energy. There is a slight difference comparing the bulk and surface La – but one has to be wary comparing bulk and surface due to self absorption.

However, the bulk shows slight band splitting with two peak contributions whereas the surface shows one peak contribution. Following the logic from the cited paper the bulk La would be less disordered than La at the surface. Which one would expect since surface terminations lead to changes in coordination compared to the bulk

Supplementary References

1. Asakura H, Shishido T, Teramura K, Tanaka T. Local structure and La L1 and L3-edge XANES spectra of lanthanum complex oxides. *Inorg Chem* 2014, **53**(12)**:** 6048-6053.

2. Aritani H, Yamada H, Yamamoto T, Tanaka T, Imamura S. XANES study of Li-MgO and Li-La2O3-MgO catalysts for oxidative coupling of methane. *Journal of Synchrotron Radiation* 2001, **8:** 593-595.
